# Retinal Toxicity in Patients Treated With Hydroxychloroquine: A Cross-Sectional Study

**Published:** 2016

**Authors:** Goldis ESPANDAR, Jamileh MOGHIMI, Raheb GHORBANI, Mohsen POURAZIZI, Mohammad-Ali SEIRI, Shervin KHOSRAVI

**Affiliations:** 1 1 Department of Ophthalmology, Semnan University of Medical Sciences, Semnan, Iran; 2 2 Ophthalmic Research Center, Labbafinejad Medical Center, Shahid Beheshti University of Medical Sciences, Tehran, Iran; 3 3 Department of Internal Medicine, School of Medicine, Semnan University of Medical Sciences, Semnan, Iran; 4 4 Research Center of Health Social Determinants, Semnan University of Medical Sciences, Semnan, Iran; 5 5 Isfahan Eye Research Center, Department of Ophthalmology, Isfahan University of Medical Sciences, Isfahan, Iran; 6 6 Students' Research Committee, Semnan University of Medical Sciences, Semnan, Iran

**Keywords:** Retinal Toxicity, Hydroxychloroquine, Autoimmune disease, Automated Perimetry

## Abstract

Hydroxychloroquine (HCQ) is an antimalarial medication that can also be used to treat autoimmune diseases. However, it can produce irreversible changes to the retina that lead to visual impairment. The aim of this study was to determine the proportion of patients treated with HCQ who develop retinal toxicity and the risk factors for the development of HCQ-induced retinal toxicity among Iranian patients. The is a cross-sectional clinical study of 59 patients who were treated with HCQ during 2014–2015. A questionnaire was used to collect data on the following demographic and clinical factors: age, gender, type of rheumatic disease, history of cataract surgery, daily and cumulative HCQ dose, and duration of HCQ use. Retinal toxicity was diagnosed on the basis of the automated perimetry results of the central 10° of vision and spectral domain optical coherence tomography. The associations between the demographic and clinical factors and retinal toxicity were assessed, and P < 0.05 was considered statistically significant. Retinal toxicity was detected in 18 (30.5%) of the patients, and 5 (8.5 %) developed color vision impairments. There was no association between retinal toxicity and sex (P = 0.514), history of cataract surgery (P = 0.479), type of rheumatic disease (P = 0.539), or daily HCQ dose (P = 0.062). However, there was a significant positive association between retinal toxicity and age (P = 0.006), cumulative HCQ dose (P = 0.002), and duration of HCQ use (P < 0.001). In conclusion, the risk factors for retinal toxicity after HCQ treatment were advanced age, use of a higher cumulative HCQ dose, and a longer duration of treatment.

## INTRODUCION

Hydroxychloroquine (HCQ) has been used for many years to reduce inflammation in the treatment of patients with multiple rheumatologic diseases, including systemic lupus erythematosus (SLE) and rheumatoid arthritis (RA) (1). Antimalarial medications (HCQ and chloroquine) are among the safest antirheumatic medications as they are rarely associated with side effects (2). The most common adverse effects are related to the gastrointestinal tract, skin, and nervous system (3).

However, one of the most serious side effects is ocular toxicity, which has been found to be more common when HCQ is used for long periods of time (4). Therefore, there is considerable concern about the risk of ocular problems among patients treated with HCQ, and regular screening (in accordance with standard guidelines) is necessary, even in the absence of ocular symptoms (5). HCQ-induced ocular toxicity can occur in two distinct areas of the eye: the cornea and the macula (5).

The changes in the macula can potentially be serious, as the consequences can include loss of vision. The mechanism by which antimalarial medications cause retinal toxicity involves the binding of the drugs to the melanin in the pigmented epithelial layer of the retina, and subsequent damage to rods and cones (6). Retinal toxicity had been classically characterized as involving bilateral “bull’s-eye” maculopathy, initial photoreceptor damage with a parafoveal distribution, and further damage with a more peripheral extramacular distribution (5).

The risk of developing retinal toxicity has been found to be dependent on the daily HCQ dose and the duration of use. The risk of retinal toxicity is <1% for those who use HCQ for up to 5 years and <2% for those who use HCQ for 5–10 years, but it rises to almost 20% after 20 years of HCQ use (5). Other major risk factors include having a concomitant renal disease and the concomitant use of certain medications such as tamoxifen (5). The findings of previous studies regarding the degree of risk of HCQ-induced retinopathy are conflicting (7).

Furthermore, although HCQ is frequently used in rheumatology, there are limited studies on the clinico-epidemiological characteristics of retinal toxicity in Iranian patients treated with HCQ (8, 9). Therefore, the aims of this study were to evaluate the percentage of Iranian patients taking HCQ who develop retinal toxicity and to identify the associated risk factors.

## MATERIALS AND METHODS

The protocol for this study was approved by the Institutional Ethics Committee of Semnan University of Medical Sciences, Semnan, Iran (project No. 584582). Following the approval of the institutional review board, we identified the medical reports of patients who were treated with HCQ between September 2014 and September 2015, and these patients were referred to an expert ophthalmologist at Semnan University of Medical Sciences.

The inclusion criteria included age ≥ 17 years and use of HCQ from September 2014 to September 2015 (along with any past use). We excluded patients if they had a clinically significant chronic ocular problem (including diabetic retinopathy) or if they were taking a medication other than HCQ with retinotoxic side effects. All 59 subjects who met the inclusion and exclusion criteria signed the informed consent forms to take part in the study. A questionnaire was used to collect patient data. The first section of the questionnaire collected data on basic demographic and clinical characteristics (age, sex, and type of rheumatic disease), and the second section collected data on the patients’ ophthalmologic history (history of cataract surgery, daily and cumulative HCQ dose, and duration of HCQ use). The rheumatic diseases were divided in to three groups: SLE, RA, or others.

Subsequently, ophthalmologic assessments were carried out for each subject. These assessments included visual acuity (VA) tests of both eyes using a Snellen chart at 6 m, a biomicroscopic examination of the fundus under dilation, a fundus examination using indirect ophthalmoscopy, a color vision exam, visual field testing of the central 10° using automated perimetry with a Swedish Interactive Threshold Algorithm (SITA)-Standard strategy, and spectral domain optical coherence tomography. The results of all the examinations were interpreted by the same ophthalmologist.

In accordance with the 2011 recommendations of the American Academy of Ophthalmology, which state that retinal toxicity should be diagnosed on the basis of alterations in at least two tests, retinal toxicity was diagnosed on the basis of the automated perimetry results of the central 10° and spectral domain optical coherence tomography (5). Although multiple methods have been suggested for retinal toxicity screening among patients treated with HCQ, there is no acceptable “gold standard” for the identification of HCQ-induced retinal toxicity before the onset of decreased vision (10-12).

The data were analyzed using Statistical Package for Social Sciences (SPSS) version 16 (SPSS Inc., Chicago, IL, USA). The data are described using frequencies, percentages, means, medians, ranges, and standard deviations. We used t-tests for numerical variables and chi-square tests for categorical variables to determine the association between retinal toxicity and sex, history of cataract surgery, type of rheumatic disease, daily HCQ dose, cumulative HCQ dose, and duration of HCQ use. P < 0.05 was considered statistically significant. Disease characteristics were compared between the two groups using chi-square for categorical variables, Mann-Whitney U test for nonparametric ordinal variables and student t test for parametric variables.

The changes from the baseline to the end of study period within each group were tested using Wilcoxon Signed Rank test for nonparametric variables and paired t test for parametric variables.

## RESULTS

The baseline characteristics of the patients are summarized in Table 1. Among the 59 included patients, 54 (91.5%) were women. They were aged 16–83 years (mean ± SD: 42.3±12.3 years). The mean duration of HCQ use ± SD was 44.5 ± 35.9 months, and the median duration was 36 months (Table 1).

The funduscopic, spectral domain optical coherence tomography and perimetry results showed that 18 (30.5%) of the patients had retinal toxicity. The patients with abnormal funduscopic values all had abnormal perimetry values. Five (8.5%) patients had abnormal color vision.

**Table 1:** Baseline Characteristics of Patients ^a^^, ^^b^

**Characteristic**	**Value**
**Sex**	
Men	5 (8.5)
Women	54 (91.5)
**Age, Y**	
Mean ± SD	42.3 ± 12.3
Range	16-83
Median	40
**Type of Rheumatic Disease**	
Rheumatoid Arthritis	36 (61)
Systemic Lupus Erythematosus	16 (27.1)
Other	7 (11.9)
**History of Cataract Surgery**	
Cataract Surgery	2 (3.4)
None	57 (96.6)
**Duration of HCQ Use**	
Mean ± SD	44.5 ± 35.9
Median	36

a Abbreviations: HCQ, hydroxychloroquine.

b Data are presented as No. (%) or Mean ± SD.

Seventeen women (31.5%) and one man (20%) had retinal toxicity, and there was no association between sex and retinal toxicity (P = 0.514). The mean age ± SD of patients with and without retinal toxicity was 45.8 ± 7.7 and 40.9 ± 13.2 years, respectively. There was a significant difference between patients aged <40 and ≥40 years in retinal toxicity (P = 0.006), with 14 (46.7%) of those aged ≥40 years and only four (13.8%) of those aged <40 years having retinal toxicity (Table 2).

Neither prior cataract surgery nor the type of rheumatic disease was associated with retinal toxicity (P = 0.479 and P = 0.539, respectively). The prevalence of retinal toxicity was 36.2% among patients who used ≤200 mg HCQ per day and 8.3% among those who used >200 mg HCQ per day, and there was no significant association between daily HCQ dose and retinal toxicity (P = 0.062). Regarding the cumulative HCQ dose, 7.4% of patients who used ≤100 g of HCQ had retinal toxicity and 52.2% of those who used >200 g had retinal toxicity (Table 2), and the cumulative HCQ dose was significantly associated with retinal toxicity (P = 0.002). The mean duration of HCQ use ± SD among patients with retinal toxicity was significantly larger than that among those without retinal toxicity (P < 0.001) at 75.2 ± 45.1 months and 29.3 ± 21.5 months, respectively (Table 2).

**Table 2:** Frequency of Retinal Toxicity in Different Groups

**Variable**	**Retinal Toxicity**		**P Value**
	**No**	**Yes**	
**Sex**			0.514
Men	4 (80)	1 (20)	
Women	38 (68.5)	17 (31.5)	
**Age, Y**			0.006
<40	25 (86.2)	4 (13.8)	
≥40	16 (53.3)	14 (46.7)	
**Type of Rheumatic Disease**			0.539
Rheumatoid Arthritis	25 (69.4)	11 (30.6)	
Systemic Lupus Erythematosus	10 (62.5)	6 (37.5)	
Other	6 (85.7)	1 (14.3)	
**Daily HCQ Dose, mg**			0.062
<200	30 (63.8)	17 (36.2)	
≥200	11 (91.7)	1 (8.3)	
**Cumulative HCQ Dose, mg**			0.002
<100	25 (92.6)	2 (7.4)	
100-199	5 (55.6)	4 (44.4)	
≥200	11 (47.8)	12 (52.2)	
**Duration of HCQ Use, mo**	29.3 ± 21.5	75.2 ± 45.1	< 0.001

a Abbreviations: HCQ, hydroxychloroquine.

b Data are presented as No. (%) or Mean ± SD.

## DISCUSSION

Our study showed that approximately 30% of the patients treated with HCQ had retinal toxicity. There was no association between retinal toxicity and sex, type of rheumatic disease, and daily HCQ dose. However, there was a positive correlation between retinal toxicity and age, cumulative HCQ dose, and duration of HCQ use. HCQ is an important medication that is very effective for treating SLE and other rheumatic conditions. It is generally well tolerated, and its side effect profile is superior to those of many other immunosuppressive medications (1). However, a major concern regarding this drug is the increased risk of developing irreversible retinopathy. Unfortunately, these retinopathies usually present insidiously (1).

Therefore, evaluations of percentages of people who take HCQ and develop retinal toxicity, and the associated risk factors, are important. The exact incidence and prevalence of HCQ-induced retinal toxicity is unknown. As the incidence and prevalence data on collagen vascular disorders (such as SLE and RA) are variable, it is difficult to calculate the exact risk of retinal toxicity among patients with RA and SLE who are treated with HCQ (13). However, the relative prevalence of retinal toxicity among those who use antimalarial medications (HCQ and chloroquine) is approximately 0.025: 100,000 (14, 15).

A registry-based study of 3,995 patients with RA or SLE who used HCQ showed that the risk of retinal toxicity was low during the first 5–7 years of HCQ use and was approximately 5 times greater after 7 years of usage (16). In another study of 2,361 patients who used HCQ continuously for at least 5 years, the overall prevalence of HCQ-induced retinopathy was 7.5% (17). The pattern and prevalence of HCQ-induced retinal toxicity differs between Iranian patients and other Asian patients, especially during the early period of usage (18), potentially due to ethnic differences.

A study by Qader et al on 60 Canadian patients who used HCQ showed that approximately 30% of the subjects showed abnormal retinal changes after 6 months. In addition, depressive changes appeared in 12 of the subjects (9). Furthermore, HCQ therapy significantly worsened the perimetric results in about 55% of the patients with abnormal anterior segments and 50.0% of subjects with abnormal posterior segments, compared with the findings at the time of enrollment (9). Further long-term large prospective studies involving different ethnic groups and highly sensitive and specific techniques for detecting early retinal changes are necessary to confirm the results of our study regarding the percentage of patients taking HCQ who develop retinal toxicity. Among the risk factors associated with retinal toxicity, the most important were the cumulative HCQ dose and duration of HCQ use. Previous research has suggested that the risk of retinal toxicity increases with an increase in the daily and cumulative HCQ doses and the duration of treatment (16, 17), but other research has indicated conflicting results regarding the association between daily HCQ dose and retinal toxicity (17). Our study indicated that there was no association with daily HCQ dose, though this may be due to the small sample size used. 

Previous research has shown that virtually all patients with retinal toxicity associated with HCQ are aged >40 years (13). The results of our study are compatible with this finding. A possible explanation for the association between age and HCQ-induced retinal toxicity is that younger individuals have significant neuronal reserves that prevent the development of retinal toxicity for many years (13). We observed that patients with SLE, RA, or other rheumatic diseases did not have different risks of retinal toxicity. Although the exact mechanism of retinal toxicity is unknown, it is likely that it is not related to autoimmunity. Previous studies have identified that the HCQ-induced damage involves outer retinal structures, retinal ganglion cells, the inner plexiform layer, and the retinal nerve fiber layer (18-21).

In conclusion, the percentage of patients with HCQ-induced retinal toxicity was high (30.5%). The risk factors were advanced age, a high cumulative HCQ dose, and a longer duration of HCQ use. Large multicenter studies using modern techniques are required to confirm the results of this study and to identify further risk factors.
